# Comorbidities and costs in HIV patients: A retrospective claims database analysis in Germany

**DOI:** 10.1371/journal.pone.0224279

**Published:** 2019-11-06

**Authors:** Stefan Christensen, Eva Wolf, Julia Altevers, Helena Diaz-Cuervo

**Affiliations:** 1 Center for Interdisciplinary Medicine (CIM) Infectious Diseases, Muenster, Germany; 2 Department of Gastroenterology and Hepatology, Muenster University Hospital, Muenster, Germany; 3 MUC Research, Munich, Germany; 4 Xcenda GmbH, Hannover, Germany; 5 Gilead Sciences Europe, Greater London, United Kingdom; University of Genoa, ITALY

## Abstract

People living with human immunodeficiency virus (PLHIV) are at high risk of developing non-HIV related comorbidities, particularly at older ages. In a retrospective claims database analysis, we compared PLHIV to a matched, non-HIV cohort to assess the prevalence of comorbidities and healthcare costs in PLHIV and the general non-HIV population in Germany. In total, 2,132 adult patients with HIV were identified in the InGef research database with HIV ICD-10 diagnosis within each year from 2011 to 2014. Of these, 1,969 could be matched to a control cohort of 3,938 individuals (1:2 ratio). Matching criteria included age, gender and socio-economic variables. The prevalence of acute renal disease (0.5% vs. 0.2%, p = 0.045), bone fractures due to osteoporosis (6.4% vs. 2.1%, p<0.001), chronic renal disease (4.3% vs. 2.4%, p<0.001), cardiovascular disease (12.8% vs. 10.4%, p = 0.006), Hepatitis B (5.9% vs. 0.3%, p<0.001) and Hepatitis C infection (8.8% vs. 0.3%, p<0.001) was significantly higher in PLHIV compared to the matched non-HIV cohort. Mean costs excluding costs for antiretroviral therapy (ART) were significantly higher in the HIV cohort (8,049€ vs. 3,658€, p<0.05). On average, PLHIV incurred excess costs of 16,441€ for ART, 2,747€ for pharmaceuticals excluding ART (p<0.05), 1,441€ for outpatient care (p<0.05) and 321€ for inpatient care (p<0.05). Devices and remedies’ costs were significantly higher in the control cohort with excess costs of 113€ (p<0.05). Considering mean total costs, excluding ART, excess costs for PLHIV amounted to 8,049€ (p<0.05). This analysis demonstrated an increased comorbidity and economic burden of PLHIV compared to matched controls. Our findings suggest that HIV remains an area of high unmet medical need. To improve patient outcomes, adequate HIV management including regular monitoring, screening for comorbidities and optimal ART selection throughout the life course of PLHIV are of key importance.

## Introduction

Over the past twenty years, highly effective antiretroviral therapy (ART) has increased the life expectancy of people living with human immunodeficiency virus (PLHIV) and has changed the standard of care from primarily an acute treatment to a long-term management of HIV [1].

PLHIV face a higher risk for non-HIV related comorbidities such as cardiovascular diseases (CVD), renal failure and bone fractures [2], particularly at older ages. In addition to age, certain ART regimens may influence or favour the progress of such comorbidities. For instance, studies have demonstrated that Tenofovir Disoproxil Fumarate (TDF) based regimens and certain protease inhibitors (PIs) are associated with decreased renal function and increased risk of major osteoporotic fracture [3].

The expected increased burden of non-communicable diseases along with a prolonged life expectancy of PLHIV entails greater use of healthcare resources, especially medications [4]. Quiros-Roldan and colleagues reported that HIV-infected patients, on average, had one or more chronic diseases in addition to HIV, compared to the general population in Italy [5]. Kidney failure, psychiatric diseases and cancer were reported as the most expensive comorbidities with substantially higher per capita costs [5].

However, data on the excess comorbidity burden in HIV-positive patients compared to the general population in Germany is scarce, as is data on the excess financial burden of HIV. Therefore, the aim of this study was to assess the prevalence of non-HIV related comorbidities and costs from the statutory health insurance (SHI) perspective among PLHIV compared to matched individuals in the general population in Germany.

## Materials and methods

### Study design

A retrospective claims database analysis was conducted to assess the prevalence of comorbidities and healthcare costs in PLHIV and the general population in Germany. Excess comorbidity and financial burden among PLHIV were evaluated using a matched cohort design.

### Data source

The InGef research database comprises claims data of approximately 4 million covered lives on an anonymized individual level. Healthcare utilization and cost of services from about 70 of the 120 health insurance providers from the SHI in Germany are included in the database. This sample represents 4.8% of the German population and 5.6% of the SHI population. It is considered representative of the general German population as it has been adjusted for age and sex to the German population. The database is also reflective of the overall German population in terms of morbidity, mortality, and drug usage [6].

### Study cohorts

The study period spanned from January 1^st^, 2011 until December 31^st^, 2014. All individuals with continuous enrolment in the InGef research database throughout the study period were eligible for inclusion in the HIV or the non-HIV cohort.

In order to investigate adult patients with a history of HIV, individuals were included into the HIV cohort if they had at least one International Statistical Classification of Diseases and Related Health Problems 10th Revision (ICD-10) German Modification (GM) code for HIV (B20, B21, B22, B23, B24) in the inpatient sector (main or secondary diagnosis) or in the outpatient sector (verified diagnosis only) within each calendar year in the study period and if they were at least 21 years old at the index quarter. The index quarter was defined as the quarter with the last available HIV diagnosis in 2014.

The control cohort from the general population comprised individuals without HIV diagnosis in the entire study period who were at least 21 years old at the index quarter but did not exceed the upper age limit, which was defined as the highest age within the HIV cohort. The index quarter in the non-HIV cohort was assigned randomly based on the distribution of the index quarters in the HIV cohort.

In order to allow for valid comparisons between the HIV cohort and the control cohort, each patient in the HIV cohort was paired with two individuals (1:2 ratio) from the non-HIV control cohort. The covariates were matched with an exact fitting at the index quarter and included age, gender, insurance status, place of residence on a district level, and the index quarter to ensure that comorbidities and costs were determined in the same quarters. Place of residence on a district level was used as a proxy for socioeconomic status (SES) as recent studies demonstrated that life expectancy in Germany varied by district with a northeast-south gradient favouring the south, primarily due to socioeconomic conditions [7–9].

Primary outcomes were stratified by the following age groups: 21–29 years, 30–39 years, 40–49 years, 50–59 years, 60–69 years, 70–79 years, and ≥80 years.

### Statistical analysis

Patient characteristics were described at the index quarter in terms of age, gender, insurance status, ART and SES group. SES was determined by dividing the residence districts into the following four median gross income groups using the Indicators, Maps and Graphics on Spatial and Urban Monitoring (INKAR) dataset [10]: SES group I with a median income from 1,910.00 Euros (€) to 2,517.00€ (85 districts), SES group II with a median income from 2,526.00€ to 3,133.00€ (242 districts), SES group III with a median income from 3,148.00€ to 3,684.00€ (62 districts), and SES group IV with a median income from 3,757.00€ to 4,371.00€ (13 districts). Baseline characteristics were compared between the cohorts using standardized differences.

Comorbidities were analysed in a one-year timeframe including the index quarter and the preceding three quarters. The most prevalent 50 comorbidities were determined via examining 3-digit-level ICD-10-GM codes in the inpatient sector (main and secondary diagnosis) or in the outpatient sector (verified diagnosis only). In addition, specific comorbidities were captured by means of ICD-10-GM or Anatomical Therapeutic Chemical (ATC) or official classification of operational procedures in Germany (OPS) codes (S1 Table): CVD, hepatitis B infection (HBV), hepatitis C infection (HCV), acute renal disease, chronic renal disease, bone fractures (wrist, shoulder, hip, spine), bone fractures due to osteoporosis (wrist, shoulder, hip, spine), primary or secondary hypertension, diabetes mellitus type 2, dyslipidaemia, and alcohol abuse. Prevalence of comorbidities was compared between the HIV cohort and the matched non-HIV cohort using the Chi-squared test.

Healthcare costs were analysed within a one-year timeframe including the index quarter and the preceding three quarters. Costs were determined in total as well as stratified by the healthcare sector and compared using the Mann-Whitney-Wilcoxon test. Level of significance was p<0.05.

## Results

### Study cohorts

In total, 2,132 HIV patients were identified in the InGef research database with an HIV diagnosis within each calendar year from 2011 until 2014 and who were at least 21 years old. Of these, 2,105 patients were eligible for matching as they did not have any missing information regarding the matching covariates. For 1,969 (93.5%) out of 2,105 HIV patients, two matching partners without HIV were available, resulting in a matched control cohort of 3,938 patients.

### Baseline characteristics

Before matching, patients in the HIV cohort were on average younger, with a mean age of 48.3 years compared to 52.3 years in the non- HIV cohort from the general population. The HIV cohort also had a considerably higher proportion of men than the non-HIV cohort (82.6% compared to 48.5%).

After the match and as a result of the direct matching procedure, all baseline characteristics were the same in both cohorts (Table 1). The mean age was 48.3 years and most patients were male (83.5%).

**Table 1:** Sociodemographic characteristics and HIV treatment in PLHIV and a matched non-HIV cohort.

	HIV cohort (n = 1,969)	Matched non-HIV cohort (n = 3,938)
Sex (male), n (%)	1,644 (83.5)	3,288 (83.5)
Age (year), mean (SD)	48.3 (12.2)	48.3(12.2)
Age groups (n)		
21–29	75	150
30–39	411	822
40–49	677	1354
50–59	481	962
60–69	177	354
70–79	119	238
≥ 80	29	58
Insurance status, n (%)		
Member	1,462 (74.3)	2,924 (74.3)
Family insured	82 (4.2)	164 (4.2)
Retired	425 (21.6)	850 (21.6)
SES group, n (%)		
Group I (MMI: 1,910€ - 2,517€)	89 (4.5)	178 (4.5)
Group II (MMI: 2,526€ - 3,133€)	976 (49.6)	1,952 (49.6)
Group III (MMI: 3,148€ - 3,684€)	643 (32.7)	1,286 (32.7)
Group IV (MMI: 3,757€ - 4,371€)	261 (13.3)	522 (13.3)
ART within quarter with the first observable HIV diagnosis in 2011, n (%)		
On ART	1,617 (82.1)	--
Not on ART	352 (17.9)	--
ART within quarter with the last observable HIV diagnosis in 2014, n (%)		
On ART	1,663 (84.5)	--
Not on ART	306 (15.5)	--

ART: antiretroviral therapy; MMI: median monthly income, SD: standard deviation, SES: Socioeconomic status

### Specific comorbidities

**Fig 1:**
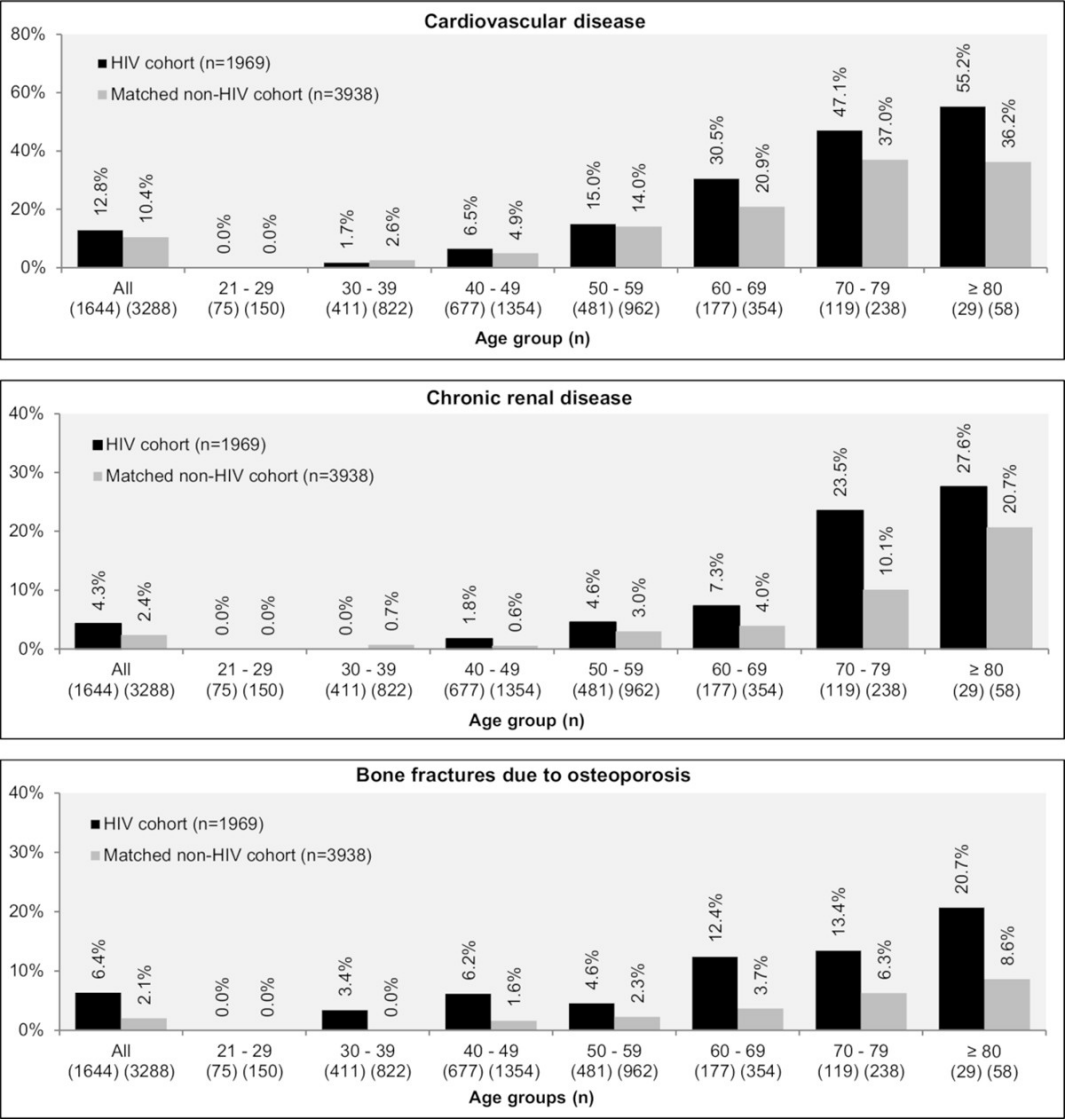
Prevalence of specific comorbidities stratified by age groups in PLHIV and a matched non-HIV cohort over the previous 2 months.

The prevalence of acute renal disease (0.5% vs. 0.2%, p = 0.045), bone fractures due to osteoporosis (6.4% vs. 2.1%, p<0.001), chronic renal disease (4.3% vs. 2.4%, p<0.001), CVD (12.8% vs. 10.4%, p = 0.006), HBV infection (5.9% vs. 0.3%, p<0.001) and HCV infection (8.8% vs. 0.3%, p<0.001) were significantly higher in the HIV cohort than in the matched non-HIV control cohort (Table 2). Prevalence of CVD, chronic renal disease and bone fractures due to osteoporosis were higher at older ages in both the HIV and matched non-HIV cohort. CVD, chronic renal disease, and bone fractures due to osteoporosis were more prevalent in the HIV cohort compared to the matched non-HIV control cohort, following a similar trend among the different age groups (Fig 1). Despite the higher CVD prevalence in HIV patients, the prevalence of hypertension (primary or secondary) was significantly higher in matched controls (29.3% vs. 32.6%, p = 0.010).

**Table 2:** Prevalence of specific comorbidities in PLHIV and a matched non-HIV cohort over the previous 12 months.

Comorbidities	HIV cohort n = 1,969 %	Matched non-HIV cohort n = 3,938 %	P value
Acute renal disease	0.5	0.2	***0*.*045***
Alcohol abuse	3.4	2.8	0.176
Bone fractures due to osteoporosis	6.4	2.1	***<0*.*001***
Cardiovascular disease	12.8	10.4	***0*.*006***
Chronic renal disease	4.3	2.4	***<0*.*001***
Diabetes mellitus (type II)	8.4	8.6	0.818
Dyslipidemia	23.9	24.0	0.914
HBV infection	5.9	0.3	***<0*.*001***
HCV infection	8.8	0.3	***<0*.*001***
Hypertension	29.3	32.6	***0*.*010***

HBV: hepatitis B virus, HCV: hepatitis C virus, P; p value for comparison between HIV and non-HIV matched control cohorts

### The 50 most common comorbidities

**Fig 2:**
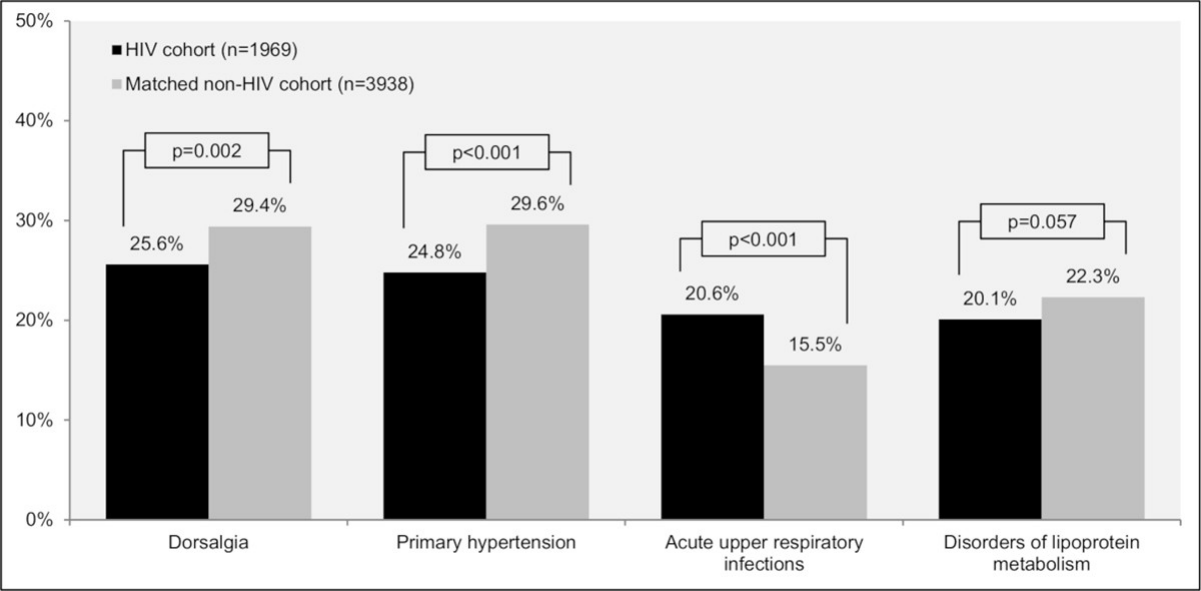
Prevalence of the 4 most common comorbidities in PLHIV and a matched non-HIV cohort over the previous 12 months.

Among the most frequent comorbidities in PLHIV and the matched non-HIV cohort were dorsalgia, primary hypertension, acute upper respiratory infections and disorders of lipoprotein metabolism, including dyslipidemia and other lipidemias. However, the prevalence of these comorbidities differed significantly in the two cohorts, except for disorders of lipoprotein metabolism (20.1% HIV vs 22.3% non-HIV, p = 0.057), with higher prevalence in the matched control group of dorsalgia (25.6% vs 29.4%, p = 0.002) and primary hypertension (24.8% vs 29.6%, p<0.001), and higher prevalence in PLHIV of acute upper respiratory infections (20.6% vs 15.5% p<0.001, Fig 2).

**Fig 3:**
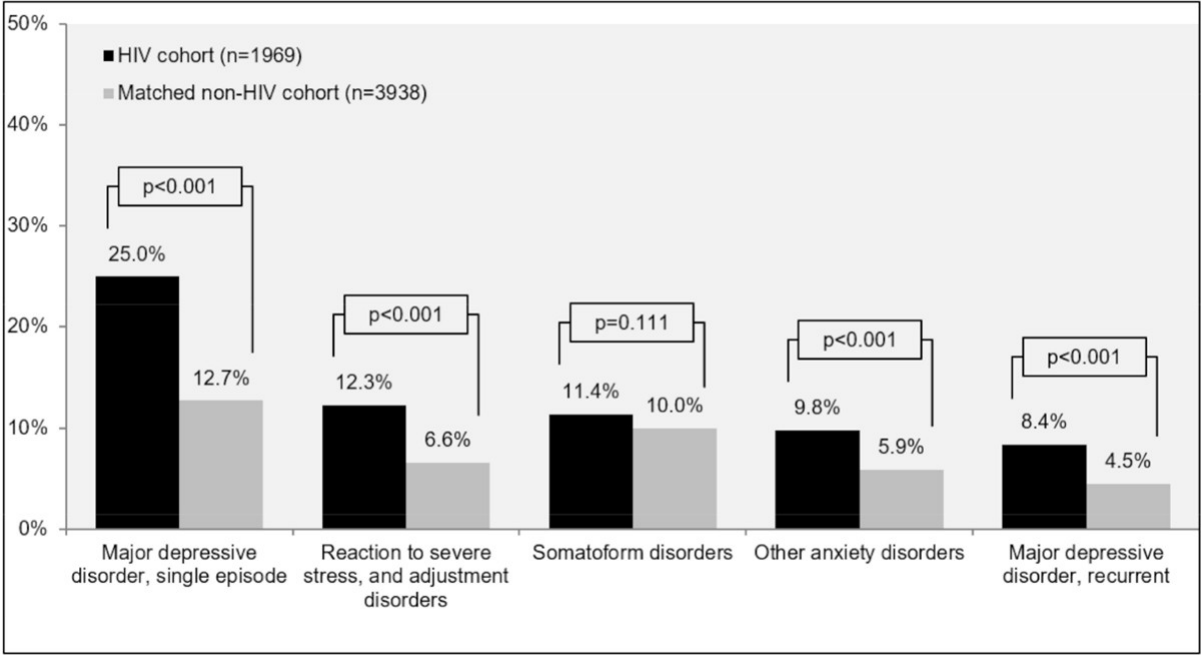
Prevalence of the 5 most common mental comorbidities in PLHIV and a matched non-HIV cohort over the previous 12 months.

Furthermore, mental disorders greatly differed between the HIV and matched non-HIV cohorts. The majority of mental illnesses and behavioural disorders such as major depressive disorder (either as a single episode or recurrent), adjustment disorders (including reaction to severe stress) and other anxiety disorders occurred significantly more often in the HIV cohort than in the matched control cohort. Additionally, somatoform disorders were observed more frequently among PLHIV than in the matched non-HIV cohort, although differences were not statistically significant (Fig 3).

### Healthcare costs

**Fig 4:**
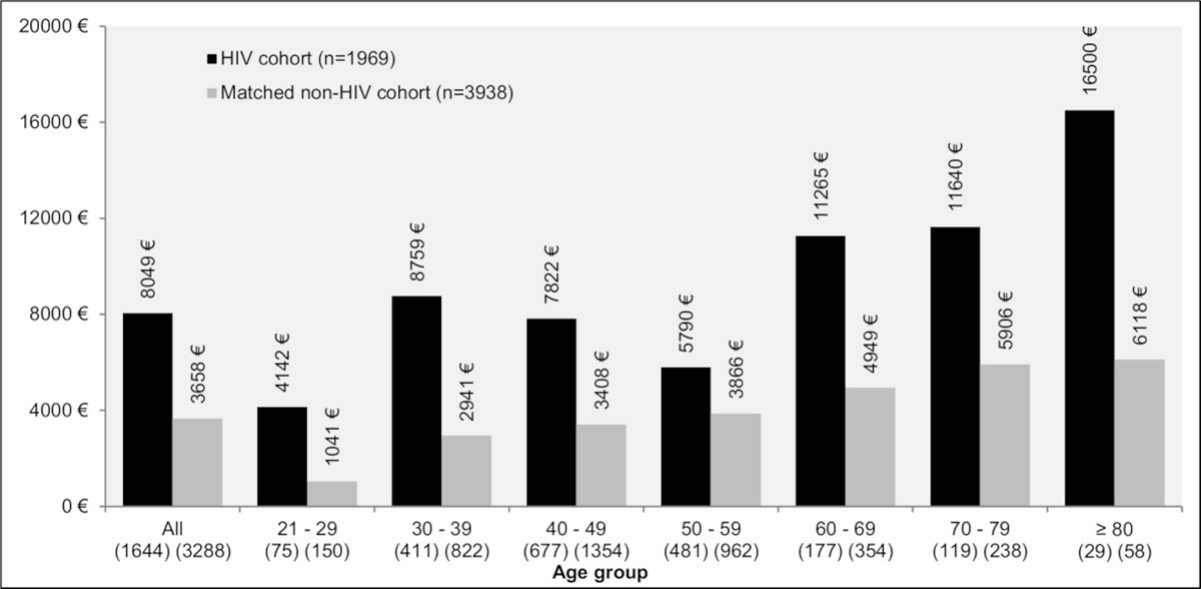
Mean total cost excluding ART in PLHIV and a matched non-HIV cohort over the previous 12 months, overall and stratified by age groups.

Table 3 presents the mean total, non-ART, healthcare costs per patient comparing the HIV cohort with the matched non-HIV cohort. In total, mean costs (not related to ART) were significantly higher in the HIV cohort than in the non-HIV matched control cohort (8,049€ vs. 3,658€, p<0.05). The same pattern was observed across age groups. In each age group, the mean costs (not related to ART) were higher in the HIV cohort compared to the non-HIV matched control cohort. In absolute numbers, PLHIV aged 80 years or older (+10,382€) had the highest excess cost. In relation to the mean costs in the control cohort, PLHIV in younger age groups (4-fold increase and 3-fold increase for PLHIV aged 21–29 years and 30–39 years, respectively) and in the oldest age group (3-fold increase for PLHIV aged 80 years or older) had the highest excess cost compared to the middle-aged groups (1 to 2-fold increase) (Fig 4).

**Table 3:** Healthcare costs by healthcare sector in PLHIV and a matched non-HIV cohort over the previous 12 months.

Costs	HIV cohort n = 1,969 Mean (Median)	Matched non-HIV cohort n = 3,938 Mean (Median)	Excess cost	P value
Medication excluding ART	3,942€ (209)	1,195€ (68)	+2,747€	***<0*.*05***
ART	16,441€ (15,041)	-	+16,441€	
Outpatient	2,147€ (1,593)	706€ (289)	+1,441€	***<0*.*05***
Inpatient	1,492€ (0)	1,171€ (0)	+321€	***<0*.*05***
Devices and remedies	294€ (0)	407€ (0)	-113€	***<0*.*05***
Sick leave payments	174€ (0)	178€ (0)	-4€	0.337
Total excluding ART	8,049€ (2,148)	3,658€ (531)	+ 4,391€	***<0*.*05***
Total including ART	24,490€ (19,328)	3,658€ (531)	+20,832€	***<0*.*05***

Costs (Euro) are reported as mean (median). Positive excess cost means that healthcare costs in HIV cohort are higher than in matched non-HIV control cohort

ART: antiretroviral treatment, P: p value for comparison between HIV and non-HIV matched control cohorts

When ART costs of 16,441€ were included, mean total costs were 24,490€ in the HIV cohort and 3,658€ in the matched control cohort, amounting to an excess cost of 20,832€ in the HIV cohort (p<0.05) (Table 3).

Aside from ART, non-ART medication costs, and outpatient and inpatient costs were the main cost drivers among HIV patients, who had significantly higher costs than the matched control cohort (Table 3). On average, PLHIV incurred excess costs of 2,747€ for pharmaceuticals excluding ART (p<0.05), 1,441€ for outpatient care (p<0.05) and 321€ for inpatient care (p<0.05). Costs for devices and remedies were significantly higher in the non-HIV control cohort, with excess costs of 113€ (p<0.05).

## Discussion

In this study, HIV patients had a higher prevalence of CVD, HBV and HCV infections, acute and chronic renal disease, bone fractures due to osteoporosis, single episode or recurrent major depressive disorders, adjustment disorders, and other anxiety disorders compared to matched patients from a representative sample of the general population in Germany. The findings of this matched cohort analysis indicated a premature onset of non-HIV related comorbidities in PLHIV, corroborating other studies [11–16]. Additionally, the current study findings extend work by Quiros-Roldan and colleagues (2016) in Italy, which suggested an increased prevalence of most chronic diseases across time, including serious psychiatric diseases [5]. A review by Nanni and colleagues (2015) further suggests a two- to four-fold increase in the prevalence of major depression compared with non-HIV infected patients [17], in line with the findings of our analysis, showing almost double the prevalence of major depressive disorders (25% HIV-cohort vs. 13% non-HIV cohort, p<0.001).

Our analysis suggests that HIV patients generally have higher CVD rates. Across age groups older than 40 years, CVD was more prevalent in PLHIV compared to the matched cohort. CVD prevalence increased markedly with age among PLHIV compared to matched controls. However, significantly fewer PLHIV presented with hypertension than their matched controls. In contrast to our findings, Triant and colleagues (2007), who compared acute myocardial infarction rates and cardiovascular risk factors in HIV-infected to non-HIV-infected individuals from two Boston healthcare facilities, found a significantly higher proportion of patients with hypertension in HIV participants compared to non-HIV [18]. Similarly, in the present study rates for diabetes mellitus and dyslipidaemia were comparable between the HIV cohort and matched control cohort, whereas Triant and colleagues found that the proportion of PLHIV having each of these comorbidities was significantly higher [18]. Different databases (registry vs. claims data), countries (Unites States of America vs. Germany), study periods (1996–2004 vs. 2011–2014) and different patient characteristics as well as the evolution of ART with fewer long-term side effects may account for these conflicting findings. Diabetes mellitus and dyslipidaemia were similar in PLHIV and matched non-HIV controls which aligns with Freiberg and colleagues’ findings (2013) that HIV itself is associated with increased risk for CVD beyond that explained by other recognized risk factors [19].

Brown and colleagues (2006) assessed the prevalence estimates of osteopenia and osteoporosis in HIV-infected individuals within a meta-analytic review. Out of 884 HIV-infected individuals, 67% had reduced bone mineral density, of whom 15% had osteoporosis. Furthermore, HIV-infected patients had 6.4-fold increased odds of reduced bone mineral density (95% confidence interval, CI, 3.7–11.3) and 3.7-fold increased odds of osteoporosis (95% CI 2.3–5.9) [20]. These findings are in line with the present study, showing a prevalence of 6.4% for bone fractures due to osteoporosis (wrist, shoulder, hip, and spine) for the HIV cohort compared to 2.1% in the control cohort.

In our study, 4.4% of the HIV cohort presented with chronic renal disease, compared to 2.4% in the matched control cohort; chronic renal disease was very common in older patients, with almost a quarter of PLHIV aged 70 and older suffering from this disease. These results support those by Althoff and colleagues (2015), who compared the risk and age at diagnosis of myocardial infarction and end-stage renal disease (ESRD) in a large sample of 97,922 patients (N = 30,243 with HIV) of the Veterans Aging Cohort Study of HIV-infected and demographically matched uninfected veterans seen between April 2003 to December 2010; while 7% of PLHIV presented with ESRD, only 5% of patients without HIV experienced ESRD (p<0.001) [21].

In the years following the study period from 2011 to 2014 included in this analysis, new treatment options for HIV have been introduced to the German market with the aim of regimen simplification and reduction of long-term toxicities including single tablet triple combinations with tenofovir alafenamide (TAF) and/or second generation integrase inhibitors such as bictegravir or dolutegravir as well as single tablet dual combinations such as dolutegravir and rilpivirine. Similar to TDF, TAF is a prodrug of tenofovir but was shown to have an improved safety profile. Multiple studies demonstrated that TAF had a reduced impact on renal function and bone mineralization in comparison to TDF [22]. The single tablet combination dolutegravir and rilpivirine was also shown to significantly improve bone mineral density and bone turnover markers when compared to TDF-based three-drug regimens [23]. In due consideration of these new treatment options, the rate of PLHIV on ART developing renal disease or osteoporosis may have decreased by now. However, this study captured comorbidities through 2014 and could form the basis and additionally guide future studies, particularly as PLHIV age and experience a higher burden of age-related comorbidities. Further studies are needed to reflect the current situation and possibly assess any potential changes in comorbidity prevalence among these patients.

Furthermore, this study demonstrated that healthcare costs unrelated to ART incurred by HIV patients in a one-year timeframe exceeded costs incurred by the matched control cohort by approximately 4,400€, with even greater differences in older age groups. These results support findings by Quiros-Roldan and colleagues (2016), who found that HIV infection ranked as the third most costly chronic disease in a North Italian population in 2014 with 9-fold increased per capita costs compared to the overall non-matched population [5].

Overall, the excess costs reported in this study potentially reflect the higher burden of comorbidities among PLHIV.

### Limitations

In general, administrative data are primarily collected for accounting purposes and not for clinical or research purposes. Thus, clinical parameters such as laboratory test results as well as the actual medication intake by the patient or the dosage of medications were not covered by the database. Also, the indication for the prescription was not captured. Lifestyle factors with a potential impact on medication use such as unhealthy diet or drug consumption were not available in the database. Additionally, other important cost categories (e.g. indirect costs such as the cost of workdays lost) are difficult to assess with claims data, as this information is only available for health insurance members but not for family members that are covered free of charge by the health insurance policy. As the data was from a SHI perspective, private sector data was unavailable, which could have resulted in an underestimation of the (excess) costs.

In the matching process, it cannot be ruled out that unobserved variables were unequally distributed in both groups, leading to biased results. In addition, since adequate matches could not be found for 6.5% of the eligible HIV patients, the study results might be not representative of the entire population with HIV. Furthermore, place of residence on a district level might be an inadequate proxy for SES, since it is an aggregate measure and does not necessarily reflect individual SES. Moreover, PLHIV have a higher risk behaviour regarding sexually transmitted diseases compared to the general population, which was not considered in the matching and therefore may lead to biased results in terms of costs [24–26].

## Conclusions

This retrospective German claims data analysis demonstrated the increased comorbidity and economic burden of PLHIV compared to individuals from the general population with similar characteristics. PLHIV faced a higher risk of CVD, HBV and HCV infections, acute and chronic renal disease, bone fractures due to osteoporosis, and mental disorders, with an earlier onset of these conditions in PLHIV.

The advanced treatment options of ARTs improve the life expectancy of PLHIV. However, when comparing HIV patients to a cohort of patients of similar characteristics not affected by HIV, these patients seem to be more prone to age-related comorbidities.

Our findings suggest that HIV remains an area of high unmet medical need demanding further research on disease management in aging populations. To improve patient outcomes and reduce the economic burden of HIV, it is crucial to better understand how the selection of ART can have an impact on comorbidities and how to better manage comorbidities throughout the life course of PLHIV.

## Supporting information

S1 Table Codes for the identification of comorbidities. (DOCX)

## References

[1] Vogel M, Schwarze-Zander C, Wasmuth JC, Spengler U, Sauerbruch T, Rockstroh JK. The treatment of patients with HIV. Dtsch Arztebl Int. 2010;107(28–29):507–16. 10.3238/arztebl.2010.0507 20703338 PMC2915483

[2] Guaraldi G, Zona S, Menozzi M, Carli F, Bagni P, Berti A, et al Cost of noninfectious comorbidities in patients with HIV. Clinicoecon Outcomes Res. 2013;5:481–8. 10.2147/CEOR.S40607 24098086 PMC3789842

[3] Bedimo R, Maalouf NM, Zhang S, Drechsler H, Tebas P. Osteoporotic fracture risk associated with cumulative exposure to tenofovir and other antiretroviral agents. AIDS. 2012;26(7):825–31. 10.1097/QAD.0b013e32835192ae 22301411

[4] Smit M, Brinkman K, Geerlings S, Smit C, Thyagarajan K, Sighem A, et al Future challenges for clinical care of an ageing population infected with HIV: a modelling study. Lancet Infect Dis. 2015;15(7):810–8. 10.1016/S1473-3099(15)00056-0 26070969 PMC4528076

[5] Quiros-Roldan E, Magoni M, Raffetti E, Donato F, Scarcella C, Paraninfo G, et al The burden of chronic diseases and cost-of-care in subjects with HIV infection in a Health District of Northern Italy over a 12-year period compared to that of the general population. BMC Public Health. 2016;16(1):1146 10.1186/s12889-016-3804-4 27829390 PMC5103392

[6] Andersohn F, Walker J. Characteristics and external validity of the German Health Risk Institute (HRI) Database. Pharmacoepidemiol Drug Saf. 2016;25(1):106–9. 10.1002/pds.3895 26530279

[7] Kuhn J, Zirngibl A, Wildner M, Caselmann WH, Kerscher G. Regional mortality differences in Bavaria. Gesundheitswesen. 2006;68(8–9):551–6. 10.1055/s-2006-926988 17039434

[8] Latzitis N, Sundmacher L, Busse R. Regional differences in life expectancy in Germany at county levels and their possible determinants. Gesundheitswesen. 2011;73(4):217–28. 10.1055/s-0030-1252035 20560119

[9] von Gaudecker HM. Regionale Mortalitätsunterschiede in Baden-Württemberg (Regional mortality differences in Baden-Wuerttemberg) 2004 [01-24-2017]. Available from: https://ub-madoc.bib.uni-mannheim.de/283/1/mea01.pdf.

[10] Bundesinstitut für Bau-, Stadt- und Raumforschung (BBSR; Federal Institute for Research on Building, Urban Affairs and Spatial Development). Indikatoren und Karten zur Raum- und Stadtentwicklung (INKAR; Indicators, Maps and Graphics on Spatial and Urban Monitoring) 2016 [05-12-2016]. Available from: http://www.inkar.de/Default.

[11] Goulet JL, Fultz SL, Rimland D, Butt A, Gibert C, Rodriguez-Barradas M, et al Aging and infectious diseases: do patterns of comorbidity vary by HIV status, age, and HIV severity? Clin Infect Dis. 2007;45(12):1593–601. 10.1086/523577 18190322 PMC3687553

[12] Friis-Moller N, Reiss P, Sabin CA, Weber R, Monforte A, El-Sadr W, et al Class of antiretroviral drugs and the risk of myocardial infarction. N Engl J Med. 2007;356(17):1723–35. 10.1056/NEJMoa062744 17460226

[13] Guaraldi G, Orlando G, Zona S, Menozzi M, Carli F, Garlassi E, et al Premature age-related comorbidities among HIV-infected persons compared with the general population. Clin Infect Dis. 2011;53(11):1120–6. 10.1093/cid/cir627 21998278

[14] Luther VP, Wilkin AM. HIV infection in older adults. Clin Geriatr Med. 2007;23(3):567–83, vii. 10.1016/j.cger.2007.02.004 17631234

[15] Powderly WG. Non-AIDS-defining illnesses in the mature patient. The graying of an epidemic—Clinical considerations of HIV and aging. 2010;3.

[16] Vance DE. Aging with HIV: clinical considerations for an emerging population. Am J Nurs. 2010;110(3):42–9. 10.1097/01.NAJ.0000368952.80634.42 20179457 PMC4571195

[17] Nanni MG, Caruso R, Mitchell AJ, Meggiolaro E, Grassi L. Depression in HIV infected patients: a review. Curr Psychiatry Rep. 2015;17(1):530 10.1007/s11920-014-0530-4 25413636

[18] Triant VA, Lee H, Hadigan C, Grinspoon SK. Increased acute myocardial infarction rates and cardiovascular risk factors among patients with human immunodeficiency virus disease. J Clin Endocrinol Metab. 2007;92(7):2506–12. 10.1210/jc.2006-2190 17456578 PMC2763385

[19] Freiberg MS, Chang CC, Kuller LH, Skanderson M, Lowy E, Kraemer KL, et al HIV infection and the risk of acute myocardial infarction. JAMA Intern Med. 2013;173(8):614–22. 10.1001/jamainternmed.2013.3728 23459863 PMC4766798

[20] Brown TT, Qaqish RB. Antiretroviral therapy and the prevalence of osteopenia and osteoporosis: a meta-analytic review. AIDS. 2006;20(17):2165–74. 10.1097/QAD.0b013e32801022eb 17086056

[21] Althoff KN, McGinnis KA, Wyatt CM, Freiberg MS, Gilbert C, Oursler KK, et al Comparison of risk and age at diagnosis of myocardial infarction, end-stage renal disease, and non-AIDS-defining cancer in HIV-infected versus uninfected adults. Clin Infect Dis. 2015;60(4):627–38. 10.1093/cid/ciu869 25362204 PMC4318916

[22] Ray AS, Fordyce MW, Hitchcock MJM. Tenofovir alafenamide: A novel prodrug of tenofovir for the treatment of Human Immunodeficiency Virus. Antiviral Research. 2016;125:63–70. 10.1016/j.antiviral.2015.11.009 26640223

[23] McComsey GA, Lupo S, Parks D, Coronado Poggio M, De Wet J, Kahl LP, et al Switch from tenofovir disoproxil fumarate combination to dolutegravir with rilpivirine improves parameters of bone health. AIDS. 2018;32:477–85. 10.1097/QAD.0000000000001725 29239893 PMC5802259

[24] Centers for Disease Control and Prevention. Behavioral and Clinical Characteristics of Persons Receiving Medical Care for HIV Infection—Medical Monitoring Project, United States, 2013 Cycle (June 2013—May 2014). HIV Surveillance Special Report 16. 2016.

[25] Blackwell DL, Lucas JW, Clarke TC. Summary health statistics for U.S. adults: National Health Interview Survey, 2012. Vital Health Stat. 2014;10(260). 24819891

[26] United Nations Office on Drugs and Crime. World Drug Report 2014: United Nations publication (Sales No. E.14.XI.7); 2014.

