# Supplementary material for: Comorbidities and costs in HIV patients: A retrospective claims database analysis in Germany
Source: PLoS One. 2019 Nov 6;14(11):e0224279. doi: 10.1371/journal.pone.0224279 (PMC6834270; doi:10.1371/journal.pone.0224279)
Supplement: S1 Table — (DOCX) [file pone.0224279.s001.docx]

**S1 Table:** Codes for the identification of comorbidities

| **Cardiovascular disease** | ICD-10-GM | - - - I21.- Acute myocardial infarction     - I22.- Recurrent myocardial infarction     - I63.- Ischemic stroke     - I70.2.- Atherosclerosis of arteries of the extremities     - I73.9 Peripheral vascular disease, unspecified     - I74.2 Embolism and thrombosis of arteries of the upper extremities     - I74.3 Embolism and thrombosis of arteries of the lower extremities     - I74.4 Embolism and thrombosis of arteries of extremities, unspecified     - I74.5 Embolism and thrombosis of iliac artery     - I80.1 Thrombosis, phlebitis or thrombophlebitis of the femoral vein     - I80.2.- Thrombosis, phlebitis or thrombophlebitis of other deep veins of the lower extremities     - I80.81 Thrombosis, phlebitis or thrombophlebitis of deep veins of the upper extremities |
| --- | --- | --- |
|  | ATC | - - - C01.- Heart therapy     - B01A* Antithrombotic agents |
|  | OPS | - - - 5-361 Insertion of an aortocoronary bypass     - 5-362 Insertion of an aortocoronary bypass using minimally invasive techniques     - 5-363.1 Other revascularization of the heart, Revision of an aortocoronary bypass     - 5-363.2 Other revascularization of the heart, Insertion of a new aortocoronary bypass     - 8-837.0 Percutaneous transluminal vascular intervention of the heart and coronary vessels, Angioplasty (balloon)     - 8-837.1 Percutaneous transluminal vascular intervention of the heart and coronary vessels, Laser-angioplasty     - 8-837.k Percutaneous transluminal vascular intervention of the heart and coronary vessels, Insertion of a non-drug-eluting stent     - 8-837.m Percutaneous transluminal vascular intervention of the heart and coronary vessels, Insertion of a drug-eluting stent     - 8-837.p Percutaneous transluminal vascular intervention of the heart and coronary vessels, Insertion of a non-drug-eluting covered stent (stent graft)     - 8-837.k Percutaneous transluminal vascular intervention of the heart and coronary vessels, Blade angioplasty (scoring- or cutting balloon)     - 8-837.u Percutaneous transluminal vascular intervention of the heart and coronary vessels, Insertion of a non-drug-eluting bifurcation stent     - 8-837.v Percutaneous transluminal vascular intervention of the heart and coronary vessels, Insertion of a drug-eluting bifurcation stent     - 8-837.w Percutaneous transluminal vascular intervention of the heart and coronary vessels, Insertion of a coated stent |
| **Hepatitis B infection** | ICD-10-GM | - - - B16.- Acute viral hepatitis B     - B18.0 Chronic viral hepatitis B with delta-agent     - B18.1 Chronic viral hepatitis B without delta-agent |
| **Hepatitis C infection** | ICD-10-GM | - - - B17.1 Acute viral hepatitis C     - B18.2 Chronic viral hepatitis C |
|  | ATC | - - - J05AB04 Ribavirin     - J05AE11 Telaprevir     - J05AE12 Boceprevir     - L03AB10 Peginterferon alfa-2b     - L0AB11 Peginterferon alfa-2a |
| **Acute renal disease** | ICD-10-GM | - - - N17.- Acute kidney failure |
|  | OPS | - - - 8-854 Hemodialysis     - 8-855 Hemodiafiltration     - 8-857 Peritoneal dialysis     - 5-555 Kidney transplant   Note: Only in absence of prior chronic renal disease diagnosis |
| **Chronic renal disease** | ICD-10-GM | - - - N18.- Chronic kidney disease |
| **Bone fractures (wrist, shoulder, hip, spine)** | ICD-10-GM | - - - M84.31 Stress fracture, unspecified site, shoulder     - M84.33 Stress fracture, unspecified site, lower arm incl. wrist     - M84.35 Stress fracture, unspecified site, pelvis and femur incl. hip     - M84.38 Stress fracture, unspecified site, other incl. spine     - M48.4.- Fatigue fracture of vertebra |
| **Bone fractures due to osteoporosis (wrist, shoulder, hip, spine)** | ICD-10-GM | - - - M80.01 Post-menopausal osteoporosis with current pathological fracture, shoulder     - M80.03 Post-menopausal osteoporosis with current pathological fracture, lower arm incl. wrist     - M80.05 Post-menopausal osteoporosis with current pathological fracture, pelvis and femur incl. hip     - M80.08 Post-menopausal osteoporosis with current pathological fracture, other incl. spine     - M80.11 Osteoporosis with current pathological fracture after ovariectomy, shoulder     - M80.13 Osteoporosis with current pathological fracture after ovariectomy, lower arm incl. wrist     - M80.15 Osteoporosis with current pathological fracture after ovariectomy, pelvis and femur incl. hip     - M80.18 Osteoporosis with current pathological fracture after ovariectomy, other incl. spine     - M80.21 Osteoporosis due to inactivity with current pathological fracture, shoulder     - M80.23 Osteoporosis due to inactivity with current pathological fracture, lower arm incl. wrist     - M80.25 Osteoporosis due to inactivity with current pathological fracture, pelvis and femur incl. hip     - M80.28 Osteoporosis due to inactivity with current pathological fracture, other incl. spine     - M80.31 Osteoporosis with current pathological fracture following malabsorption after surgery, shoulder     - M80.33 Osteoporosis with current pathological fracture following malabsorption after surgery, lower arm incl. wrist     - M80.35 Osteoporosis with current pathological fracture following malabsorption after surgery, pelvis and femur incl. hip     - M80.38 Osteoporosis with current pathological fracture following malabsorption after surgery, other incl. spine     - M80.41 Drug-induced osteoporosis with current pathological fracture, shoulder     - M80.43 Drug-induced osteoporosis with current pathological fracture, lower arm incl. wrist     - M80.45 Drug-induced osteoporosis with current pathological fracture, pelvis and femur incl. hip     - M80.48 Drug-induced osteoporosis with current pathological fracture, other incl. spine     - M80.51 Idiopathic osteoporosis with current pathological fracture, shoulder     - M80.53 Idiopathic osteoporosis with current pathological fracture, lower arm incl. wrist     - M80.55 Idiopathic osteoporosis with current pathological fracture, pelvis and femur incl. hip     - M80.58 Idiopathic osteoporosis with current pathological fracture, other incl. spine     - M80.81 Other osteoporosis with current pathological fracture, shoulder     - M80.83 Other osteoporosis with current pathological fracture, lower arm incl. wrist     - M80.85 Other osteoporosis with current pathological fracture, pelvis and femur incl. hip     - M80.88 Other osteoporosis with current pathological fracture, other incl. spine     - M80.91 Unspecified osteoporosis with current pathological fracture, shoulder     - M80.93 Unspecified osteoporosis with current pathological fracture, lower arm incl. wrist     - M80.95 Unspecified osteoporosis with current pathological fracture, pelvis and femur incl. hip     - M80.98 Unspecified osteoporosis with current pathological fracture, other incl. spine |
|  | ATC | - - - M05BA Bisphosphonates     - M05BB Bisphosphonates, combinations     - A11CC Vitamin D and analogs     - A12A Calcium |
| **Hypertension** | ICD-10-GM | - - - I10.- Essential (primary) hypertension     - I15.- Secondary hypertension |
|  | ATC | - - - C02 Antihypertensives     - C03 Diuretics     - C07 Beta adrenergic receptor antagonist     - C09 Agents acting on the renin-angiotensin-aldosterone system |
| **Diabetes type 2** | ICD-10-GM | - - - E11.- Type 2 diabetes mellitus |
|  | ATC | - - - A10 Antidiabetics EXCL. those who have a type 1 diabetes mellitus diagnosis (E10.-) in the same quarter as the prescription date |
| **Dyslipidemia** | ICD-10-GM | - - - E78.- Disorders of lipoprotein metabolism and other lipidemias |
|  | ATC | - - - C10 Agents acting on lipid metabolism |
| **Alcohol abuse** | ICD-10-GM | - - - F10 Alcohol related disorders |
|  | ATC | - - - N07BB Agents for the treatment of alcohol dependency |

ATC: Anatomical Therapeutic Chemical, OPS: Official classification of operational procedures in Germany, ICD-10-GM: International Statistical Classification of Diseases and Related Health Problems10th Revision German Modification
